# The pathophysiological role of acute inflammation after spinal cord injury

**DOI:** 10.1186/s41232-016-0026-1

**Published:** 2016-10-17

**Authors:** Seiji Okada

**Affiliations:** 1 Department of Advanced Initiatives, Graduate School of Medical Sciences, Kyushu University, 3-1-1 Maidashi, Higashi-ku, Fukuoka, 812-8582 Japan; 2 Orthopaedics, Graduate School of Medical Sciences, Kyushu University, Fukuoka, Japan

**Keywords:** Spinal cord injury, Neutrophil, Microglia

## Abstract

Traumatic spinal cord injury (SCI) causes irreparable severe motor and sensory dysfunction. Mechanical trauma rapidly leads to blood-spinal cord barrier disruption, neural cell death, axonal damage, and demyelination, followed by a cascade of secondary injury that expands the additional inflammatory reaction at the lesion site. Although the role of inflammation in this phase is complex, a number of studies have suggested that inflammatory responses spread the damage to the surrounding tissue, induce apoptotic cell death, and impair spontaneous regeneration and functional recovery. However, recent advances in experimental technology, such as the depletion antibodies for a specific fraction of inflammatory cells and the genetically engineered mice deficient only in specific cells, suggest the beneficial aspects of inflammatory cells, such as a neuroprotective effect, the removal of cellular debris, and the attenuation of the inflammatory reaction in general. In this review, I summarize our recent findings about the biological role of inflammatory cells, especially infiltrating neutrophils and activated microglia after SCI. A better understanding of the pathophysiological role of inflammation in the acute phase of SCI will aid in the development of therapeutic strategy to enhance the functional recovery after SCI.

## Background

Traumatic spinal cord injury (SCI) is a major public health problem and a devastating event for individuals that causes permanent severe motor/sensory dysfunction and significantly degrades the quality of life. SCI is known to result in neurological deficits through both the primary and secondary damage. The “primary” injury encompasses the immediate mechanical damage to the spinal cord tissue that occurs at the moment of impact, which is irreversible and not preventable. The “secondary” injury, by contrast, is incurred as a result of the pathological processes initiated at the time of the primary injury and continues for several days or months after injury and is amenable to therapy.

## Main text

### Inflammatory reaction and the secondary injury

In the secondary injury process of SCI, the infiltration of leukocytes and activation of glial cells can aggravate tissue damage by releasing proteases, reactive oxygen intermediates, lysosomal enzymes, and proinflammatory cytokines/chemokines [1, 2]. Although the role of inflammation in this phase is complex, with certain beneficial aspects as well, such as the removal of cellular debris, a number of studies have suggested that inflammatory responses spread the damage to surrounding tissue, induce apoptotic cell death, and impair spontaneous regeneration and functional recovery [3]. To protect the injured spinal cord from these secondary pathological processes, several approaches to manipulate the inflammatory responses have been assessed and found effective. These approaches include the blockage or neutralization of specific cytokine signaling using a monoclonal antibody, the delivery of anti-inflammatory drugs, and the use of genetically modified animals. Indeed, we previously examined whether or not the administration of IL-6 receptor antibody immediately after SCI attenuated the secondary injury and caused a therapeutic effect, since IL-6 is a principal proinflammatory cytokine in SCI [4].

IL-6 signaling plays roles in regulating various steps in inflammatory reactions, such as the activation and infiltration of neutrophils, monocytes, macrophages, and lymphocytes. Indeed, previous studies from other research groups have reported that the delivery of the IL-6/sIL-6R fusion protein to spinal cord injury sites induced a sixfold increase in neutrophils and a twofold increase in macrophages and microglial cells and expanded the damaged area [5]. We therefore speculated that blockage of IL-6 signaling would suppress the inflammatory response and ameliorate the secondary injury after SCI. We found that the number of infiltrated macrophages as well as scar tissue formation was significantly reduced, resulting in improved functional recovery [4]. The same strategy conducted later by other groups also demonstrated that the temporary inhibition of IL-6 signaling reduced the infiltration of hematogenous macrophages and the activation of the phagocytic activity of microglial cells [6, 7]. In addition to the anti-inflammatory effect, this approach also had a number of additional effects, including the attenuation of glial scar formation and preservation of neuroprotective phosphatidylcholine [8]. Moreover, a clinical merit of this strategy is that humanized antibody to human IL-6R (ACTEMRA®, tocilizumab) has already been in widespread use for rheumatoid arthritis and its efficacy as well as safety profile was confirmed.

However, in contrast to these reports, IL-6 itself was reported to enhance spinal cord repair by modifying the migration of reactive astrocytes or enhancing axonal regrowth [9, 10]. Although these results seem inconsistent, this contributes to the consequence of the context-dependent pleiotropic actions of IL-6 in SCI. During the acute phase of SCI, IL-6 family cytokines act primarily as potent proinflammatory mediators and cause secondary injury but also enhance the repair process after the subacute phase of SCI. These findings for IL-6 signaling suggest that the inflammatory response in SCI is very complicated and has context-dependent pleiotropic actions.

### Flow cytometric evaluation of infiltrating leukocytes in SCI

**Fig. 1:**
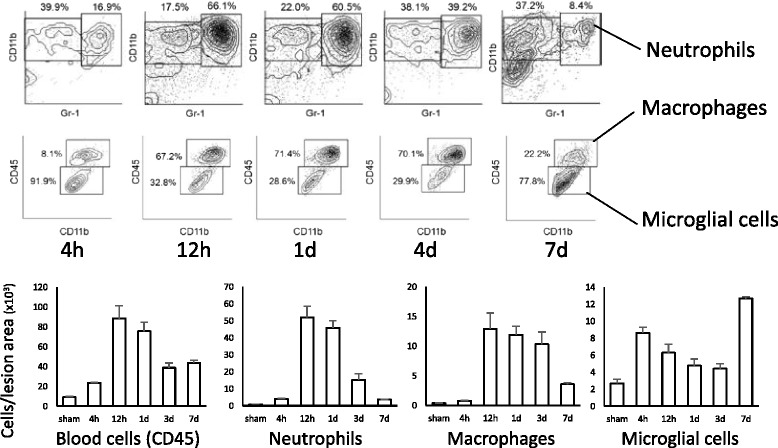
Time course of infiltrating inflammatory cells in injured spinal cord. A quantitative time course evaluation of the infiltrated neutrophils (CD45^+^CD11b^+^Gr-1^+^ fraction), macrophages (CD11b^+^Gr-1^−^CD45^high^), and microglial cells (CD11b^+^Gr-1^−^CD45^int^) in the SCI mice (Th9 contusion injury, 70 kdyn). Dot plots and graph data (*n* = 6 in each time point) were quoted from [13]

In the research field of SCI, the conventional evaluation of inflammatory cell infiltration has been mainly limited to histological analyses. However, accurate quantification with histology is relatively difficult, as the lesion site is too fragile to treat in the acute phase of injury when the most prominent cell infiltration is observed. We therefore have induced flow cytometry, which enables the accurate detection and direct isolation of these cells for the evaluation of inflammatory cells after SCI [11]. With this method, we were able to quantitatively examine the detailed profile of infiltrated leukocytes into the lesion area (Fig. 1). The infiltrated neutrophil population had increased dramatically 12 h after SCI and remained at a high level for up to 1 day before gradually decreasing thereafter. Although the peak monocyte/macrophage infiltration is commonly understood to occur at a later phase than neutrophil infiltration, including in human SCI [12–14], we found that that monocyte/macrophage infiltration also peaked at 12 h after SCI. In addition, the temporal change in the number of infiltrated monocytes/macrophages was completely different from that of the microglial cells, which dramatically increased at 7 days after SCI. We attribute this discrepancy between the present and previous reports to the shortcomings of the immunohistological analyses, which have difficulty in discriminating infiltrated monocytes/macrophages from resident microglial cells. This methodology enables us to quantify not only the accurate number of the cells at multiple time points after SCI but also the secretory activity of the inflammatory mediators by sorting the inflammatory cell fractions [11].

### Modulation of infiltrating neutrophils after SCI

Among the infiltrating leukocytes in the acute phase of SCI, neutrophils are considered to be one of the most potent triggers of post-traumatic spinal cord damage, which occurs through the release of proteases, reactive oxygen intermediates, nitric oxide, and lysosomal enzymes. Despite the fact that neutrophils are essential for innate immunity and important as anti-infectious factors in host defense, several studies focusing on the suppression of neutrophil infiltration have reported reduced severity of secondary injury and better functional recovery after SCI [15, 16].

The process of neutrophil infiltration to the lesion site is enhanced and amplified by variety of factors, such as proinflammatory cytokines, eiconosides, and adhesion molecules. Of these factors, leukotriene B4 (LTB4) is a highly potent lipid chemoattractant for neutrophils. LTB4 is produced rapidly by arachidonic acid cascade from membrane phospholipids without any requirement of transcription or translation and is mediated by its high-affinity specific receptor LTB4 receptor 1 (BLT1) [17]. In addition to this effect, LTB4 activates neutrophils that promote lysosomal enzyme release and superoxide production. This LTB4 biosynthesis system exerts its effect on the injured tissue faster than other inflammatory cytokines and chemokines, implying that LTB4 might have a superior influence on the inflammatory cascade [18].

Previous studies have demonstrated that LTB4 is not only an important mediator in the regulation of microbial infection but also deeply related to several inflammatory diseases, autoimmune diseases, and atherosclerosis [19–22]. However, as for traumatic injury, the physiological role of LTB4 is not yet well understood. In addition, few analyses have examined the relationship between LTB4 and pathophysiology after SCI, although LTB4 may be a major contributive factor for inflammatory cell infiltration.

We therefore analyzed the pathophysiological involvement of LTB4 in a mouse SCI model using BLT1-deficient mice. Our results showed that BLT1-knockout mice exhibited a 23 % decrease in neutrophils and 10 % decrease in macrophages after SCI compared to the wild-type mice [11]. These reduced numbers of infiltrated leukocytes resulted in the suppression of neural apoptosis, less demyelination, and reduced proinflammatory cytokine expression as well as better functional recovery in BLT1-knockout mice than in wild-type mice [11]. These results showed that the LTB4-BLT1 pathway was indeed involved in the pathogenesis of traumatic secondary damage through the amplification of neutrophils and macrophages infiltration, suggesting that neutralizing LTB4 has potential as a therapeutic strategy during the acute phase of SCI.

### Pathophysiological role of microglia in SCI

Microglial cells constitute about 10 % of the adult central nervous system (CNS) cell population and represent the innate immune system of the spinal cord. Under pathological conditions such as neurodegenerative disease, stroke, tumor invasion, and traumatic injury, these cells become activated, surround damaged and dead cells, and clear cellular debris from the area, much like the phagocytic macrophages of the immune system [23]. In healthy mammalian brain tissue, microglia display characteristically elongated cell bodies with spine-like processes that often branch perpendicularly. Although microglia were initially believed to be essentially quiescent cells, recent studies have revealed that they are continually surveying their microenvironment and represent the first line of defense against invading pathogens or other types of CNS tissue injury [24, 25]. Indeed, we found that the spinal microglial secretory activity was quickly stimulated at 3 h post SCI in response to pathological changes, while the infiltration of other leukocytes peaked at 12 h post SCI [11, 26]. In addition, we demonstrated that microglial activity was significantly attenuated in young mice compared to adult mice, with reduced leukocyte infiltration and neural damage as well as better functional recovery in the younger mice than in the older ones [26]. The expressions of potent chemoattractant for neutrophil infiltration, IL-6, and CXCL1 were also significantly reduced in the microglia isolated from the young mice. Considering that these chemoattractants are dominantly secreted by activated microglia and that microglial activation occurred prior to the infiltration of leukocytes, microglial activity appears to be critical for the trigger of propagation and enhancement of the inflammatory response. Leukocytes that infiltrate the lesion site also produce cytokines/chemokines by interaction with the other immune cells or microglial cells, leading to the amplification of the chemotactic gradient and to further infiltration of leukocytes to the lesion site [27]. We therefore believe that the reduced immediate activation of microglial cells in young mice results in the decreased infiltration of neutrophils, leading to reduced amplification/exaggeration of the inflammatory response in SCI.

Although the precise mechanisms of microglial activation remains unclear, several basic research studies have reported that hyperglycemia is involved in the activation of resident monocytic cells, including microglia. For example, the number of pancreatic resident monocytes is increased in hyperglycemic rodents, leading to the up-regulation of islet-derived inflammatory factors, such as IL-6 and IL-8 [28]. In addition, peritoneal monocytes are activated under hyperglycemic conditions, subsequently inducing a greater production of TNFα than that associated with a normoglycemic state [29]. Furthermore, hyperglycemia correlates with worsening of tactile allodynia accompanied by the hyperactivation of dorsal horn microglia [30].

**Fig. 2:**
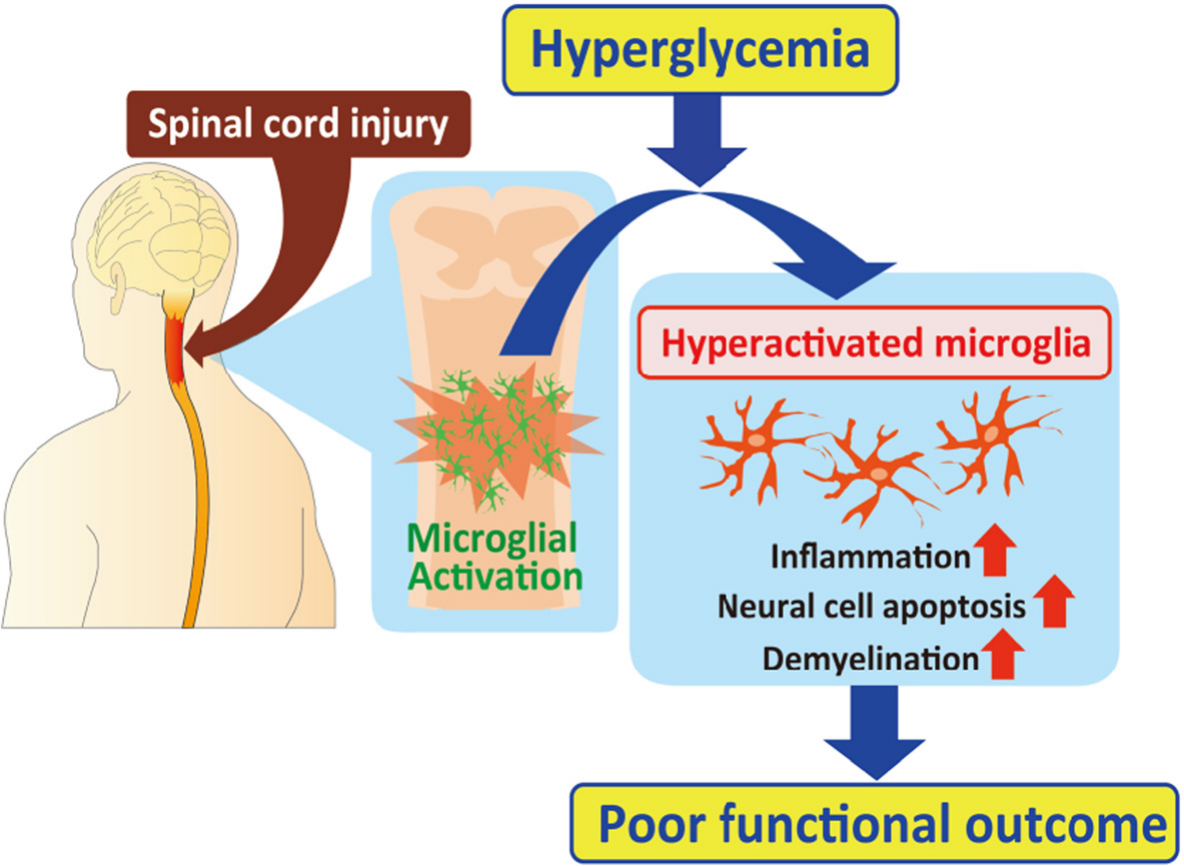
Hyperglycemia in the acute phase of SCI is associated with poor functional outcomes

Because microglial activation is associated with secondary injury after SCI, we hypothesized that hyperglycemia may also influence the pathophysiology of SCI by altering microglial responses. We thus investigated the effects of hyperglycemia on the pathophysiological processes and motor functional outcomes in two experimental mouse models of hyperglycemia in the acute phase of injury [31]. An in vivo cell type-specific gene expression analysis with flow cytometry revealed enhanced the proinflammatory reactivity in the microglial cells of the hyperglycemic mice. We found that hyperglycemia induced the overactivation of NF-kB in microglial cells as well as excessive inflammation, resulting in a poor functional recovery after SCI [31]. We also conducted a multivariable linear regression analysis of the clinical data obtained from 528 human SCI subjects, which provided entirely new evidence showing that acute phase hyperglycemia is a critical factor in the poor functional outcomes of SCI. Finally, we showed that achieving glycemic control can ameliorate the pathological and functional outcomes of hyperglycemic mice, thus supporting the existence of a direct relationship between acute hyperglycemia and the exacerbation of SCI outcomes [31] (Fig. 2).

With regard to the mechanisms involved in the hyperglycemia-related overactivation of NF-kB in microglia, NADPH oxidase is considered to possibly play a role. NADPH oxidase is present in several types of phagocytes, including microglia, causing inflammatory activation of these cells [32]. In addition, several studies have reported that hyperglycemia enhances the NADPH oxidase activity in innate immune cells [33, 34]. Furthermore, NADPH oxidase is known to produce reactive oxygen species (ROS) [35], which may promote the translocation of NF-kB [36]. Therefore, hyperglycemia may promote the translocation of NF-kB in microglial cells via the NADPH oxidase/ROS/NF-kB pathway. The fact that the increased expression of NADPH oxidase and ROS has been confirmed in spinal microglial cells after injury also supports the role of this pathway [37]. These findings shed light on the importance of achieving tight glycemic control in acute human SCI to obtain better neurological outcomes, also providing a better understanding of the inflammatory machinery after SCI.

### Resolution of acute inflammation after SCI

Although the acute inflammation after SCI spontaneously diminishes within a short period of time, the mechanism underlying this inflammatory resolution is largely unknown. Recently, we demonstrated that the infiltrating Ly6C^+^Ly6G^−^ immature monocyte fraction exhibited the same characteristics as myeloid-derived suppressor cells (MDSCs) and played a critical role in the resolution of acute inflammation and in the subsequent tissue repair after SCI [38].

**Fig. 3:**
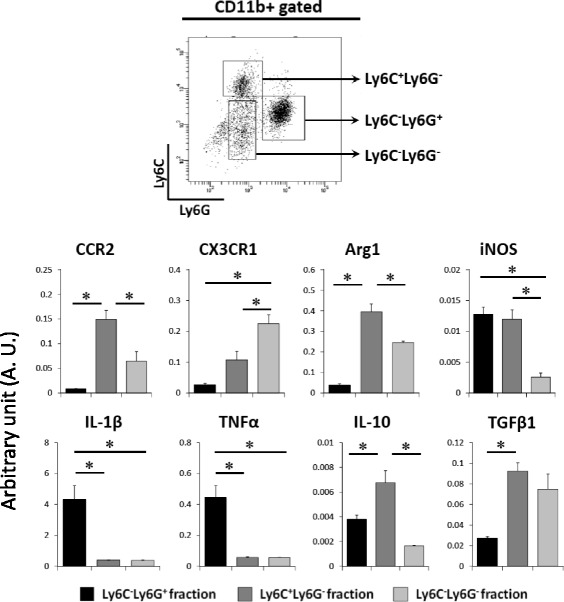
Three subsets of CD45^+^CD11b^+^ infiltrating cells and their each phenotype. Injured mice spinal cords (Th9 contusion injury, 70 kdyn) were harvested at 4 days after injury and subjected to a flow cytometric analysis. Ly6C^+^Ly6G^−^, Ly6C^−^Ly6G^+^, and Ly6C^−^Ly6G^−^ fractions were analyzed by quantitative RT-PCR. *p < 0.05 using a Kruskal–Wallis H test, with Bonferroni’s post hoc correction. Data were quoted from [38]

Immediately after SCI, a large number of CD11b^+^Gr-1^+^ inflammatory cells infiltrated the lesion area and led to the secondary damage of neural tissue. Although Gr-1 surface antigen is a common epitope on Ly6C and Ly6G, which express monocytic and granulocytic subsets, respectively, the detailed role of each subset at the lesion areas remains elusive. We therefore evaluated the temporal change in the infiltration of Ly6C^+^Ly6G^−^, Ly6C^−^Ly6G^−^, and Ly6C^−^Ly6G^+^ cell subsets in the CD45^+^CD11b^+^ fraction by flow cytometry from 4 h until 7 days after SCI [38]. The flow cytometry analysis revealed that the infiltrating Ly6C^−^Ly6G^+^ and Ly6C^+^Ly6G^−^ fractions had similar patterns of change, peaking at 12 h after injury, whereas the Ly6C^−^Ly6G^−^ fraction increased gradually with time. To investigate the physiological roles of these myeloid-derived inflammatory cell subsets after SCI, we used FACS to isolate each subset based on their expression of Ly6C and Ly6G cell surface antigens. We confirmed that the flow cytometry-sorted Ly6C-Ly6G^+^ fraction expressed significantly higher levels of CXCR1 and CXCR2, the Ly6C^+^Ly6G^−^ fraction expressed a higher level of CCR2, and the Ly6C^−^Ly6G^−^ fraction expressed higher levels of CX3CR1 than the other fractions, which indicated that each subset was regulated by different chemokines. The infiltrating Ly6C^−^Ly6G^+^ fraction showed proinflammatory properties with elevated expression of IL-1β and TNFα. In contrast, we confirmed that the Ly6C^+^Ly6G^−^ fraction had elevated expression of both iNOS and arginase 1 (Fig. 3). This expression pattern is a typical feature of MDSCs, which exert immunosuppressive effects by modulating macrophage activation toward an immunosuppressive phenotype. In addition, the Ly6C^+^Ly6G^−^ fraction had elevated expression of anti-inflammatory mediators such as IL-10, TGFβ, and VEGF, which is also consistent with the typical features of MDSCs. We also demonstrated that complete depletion of this population resulted in prolonged inflammation and significantly exacerbated tissue edema, vessel permeability, and hemorrhaging, causing impaired neurological outcomes. Furthermore, the transplantation of MDSCs at lesion areas significantly attenuated acute inflammation and promoted tissue repair, which improved neurological outcomes after SCI [38].

Among the anti-inflammatory factors from Ly6C^+^ MDSCs, IL-10 functions as a potent inducer of HO-1 in macrophages [39]. HO-1 is a heme-degrading enzyme that protects tissues from free heme toxicity. In addition, it also has a direct effect of attenuating inflammation [40]. We confirmed that transplantation of MDSCs significantly up-regulated HO-1 expression, suggesting that MDSCs created an environment favorable for tissue repair. In addition, the expression of both arginase 1 and iNOS was enhanced in the lesion areas after MDSC transplantation for 1 week after SCI [38]. This up-regulation of both arginase 1 and iNOS was a determining factor for defining the characteristics of MDSCs. These findings clarified the role of MDSCs after traumatic SCI and suggested the potential utility of an MDSC-based therapeutic strategy for the acute phase of SCI.

## Conclusions

Although inflammatory reactions lead to further damage and dysfunction after SCI, we confirmed that complete neutrophil depletion using the Gr-1 antibody severely impaired the functional recovery in a mouse SCI model. Thus, whether or not neuroinflammation after SCI has a neurotoxic or neuroprotective effect remains highly controversial. Although only minor attention has been paid to the role of inflammation in tissue protection after SCI thus far, it could be an essential factor for a well-balanced inflammatory reaction under pathological conditions. Nevertheless, more basic research should be conducted to clarify the detailed pathophysiological role of inflammation after SCI, which suggest a new approach for SCI treatment by modifying the inflammatory response in SCI.

## Abbreviations

BLT1: Leukotriene B4 receptor 1
CCR2: C-C chemokine receptor type 2
CNS: Central nervous system
CX3CR1: Chemokine (C-X3-C motif) receptor 1
CXCL1: Chemokine (C-X-C motif) ligand 1
FACS: Fluorescence activated cell sorting
HO-1: Heme oxygenase 1
IL-6: Interleukin-6
iNOS: Inducible nitric oxide synthase
LTB4: Leukotriene B4
MDSC: Myeloid-derived suppressor cells
NADPF: Nicotinamide adenine dinucleotide phosphate
NF-kB: Nuclear factor-kappa B
ROS: Reactive oxygen species
SCI: Spinal cord injury
TGFβ: Transforming growth factor β
TNFα: Tumor necrosis factor α
VEGF: Vascular endothelial growth factor
